# Dismantling structural inequities in global prevention of childhood and adolescent non-communicable diseases

**DOI:** 10.7189/jogh.16.03018

**Published:** 2026-08-07

**Authors:** Kurubaran Ganasegeran, Mohd Kamarulariffin Kamarudin, Shameni Sunasundram, Mohd Rizal Abdul Manaf, Erwin Jiayuan Khoo

**Affiliations:** 1 Clinical Research Centre, Seberang Jaya Hospital, Ministry of Health Malaysia, Seberang Perai, Malaysia; 2 Institute for Medical Research, National Institutes of Health, Ministry of Health Malaysia, Setia Alam, Malaysia; 3 Director’s Office, Seberang Jaya Hospital, Ministry of Health Malaysia, Seberang Perai, Malaysia; 4 Department of Public Health Medicine, Faculty of Medicine, Universiti Kebangsaan Malaysia, Kuala Lumpur, Malaysia; 5 Department of Paediatrics, School of Medicine, IMU University, Kuala Lumpur, Malaysia; 6 Center for Bioethics, Harvard Medical School, Harvard University, Boston, Massachusetts, USA

**Keywords:** childhood, adolescent, non-communicable disease, social determinants of health, health policy, intergenerational justice

## Abstract

Non-communicable diseases (NCDs) account for a growing proportion of morbidity and disability among children and adolescents worldwide. Contemporary NCD preventive strategies are predominantly adult-centric, resulting in delayed prevention and widening inequities, particularly in low- and middle-income countries. This neglect reflects a deeper structural and ethical failure, sustained by fragmented financing, siloed governance, commercial determinants of health, and short-term political priorities. Drawing on life–course theory, systems thinking, and principles of global health justice, childhood and adolescent NCD neglect can be understood as a systematic gap in long-term health governance that undermines human capital formation and entrenches socioeconomic disadvantage. A policy reorientation that positions early-life NCD prevention as a strategic investment in sustainable development is required. Priority reforms include embedding paediatric NCD services within universal health coverage, strengthening age-disaggregated surveillance systems, institutionalising intersectoral governance mechanisms, and establishing global accountability frameworks. Protecting children and adolescents from preventable NCDs constitutes not only a public health necessity but a moral, economic, and political imperative.

Non-communicable diseases (NCDs) are chronic, long-duration conditions arising from the interaction of genetic, physiological, environmental, and behavioural factors. In the context of childhood and adolescence, it is important to distinguish two partially overlapping categories that together constitute the paediatric NCD burden [1]. The first comprises established paediatric NCDs: conditions with onset or significant clinical manifestation in childhood and adolescence, including type 1 diabetes, childhood cancers, asthma, congenital heart disease, and mental health disorders. The second comprises NCD risk factor trajectories: behavioural, metabolic, and environmental exposures that originate in early life and powerfully determine adult NCD risk, including obesity, dyslipidaemia, hypertension, tobacco initiation, and poor nutrition. Both categories are addressed in this paper, and the distinction matters for policy design: the former demands integrated treatment and transitional care, while the latter demands primary prevention and structural reform.

The burden of NCDs among children and adolescents varies globally. Despite significant long-term declines in NCD-related mortality, disability driven by NCDs continues to rise. Data from the Global Burden of Disease (GBD) Study 2019 indicate that NCDs account for 49.8% of total disability-adjusted life years (DALYs) in South-East Asia and 65.1% in the Western Pacific region among adolescents aged 10–24 years, with neoplasms, cardiovascular diseases, and mental disorders as the leading contributors across 42 countries [2]. In the EU, overall NCD years lived with disability among this age group increased by 1.44% between 1990 and 2019, with the largest growth observed in diabetes and kidney diseases (37.8%), driven primarily by an 80.5% increase in diabetes years lived with disability [2]. NCD death and DALY rates decrease consistently with increases in the sociodemographic index and universal health coverage effective coverage index [2], highlighting the equity dimension of this burden. Behavioural risk factors, such as alcohol consumption, drug use, poor diet, and childhood abuse, show stark gender and regional disparities that drive this burden [1,3]. In 2021, behavioural risk factors contributed to an estimated 9.0 million DALYs from NCDs globally among adolescents and young adults aged 10–24, with substance use disorders and mental disorders as the leading contributors [3]. Commercial determinants compound these risks, as large corporations in the ultra-processed food and beverage, tobacco, and digital entertainment industries market harmful products to babies [4], children, and adolescents [5,6]. Importantly, these industries are not homogeneous: their health impacts differ considerably in kind, magnitude, and governance response. Tobacco represents the most comprehensively regulated industry, whereas alcohol and ultra-processed food manufacturers are often identified as potential partners in multisectoral health initiatives. Some sectors have shown partial responsiveness to regulatory pressure, for example, through product reformulation under front-of-pack labelling schemes, whereas others, particularly infant formula marketers and digital food advertisers targeting children, have demonstrated systematic resistance to public health regulation. This variation argues for sector-specific regulatory strategies rather than uniform approaches.

Traditional public health efforts for paediatric populations have focused predominantly on infectious diseases and acute conditions, sidelining NCDs [1,7]. National NCD strategies remain adult-centric, contributing to data gaps, delaying early interventions, and entrenching health inequities [8,9].

## THE HIDDEN COSTS: HUMAN CAPITAL LOSS AND WIDENING INEQUITIES

Child and adolescent NCDs and their risk factor trajectories impose massive economic tolls with far-reaching macroeconomic implications. In high-income nations, managing childhood obesity alone could exceed USD 10 billion annually [10]. Indirect costs, however, constitute the greatest burden. For example, in childhood asthma, school absenteeism and lost caregiver productivity drive much of the impact [11]. Compounded by profound psychosocial strain, social disruption, and stigma experienced by young people and their families [11], these losses are part of a broader NCD economic crisis. The World Economic Forum and Harvard School of Public Health [12] estimated that leaving cardiovascular disease, chronic respiratory disease, cancer, diabetes, and mental health conditions unaddressed could cost the global economy USD 47 trillion in output between 2010 and 2030 [13], a figure equivalent to 75% of global gross domestic product in 2010. It is important to note that this estimate encompasses the full adult and paediatric NCD spectrum. Nevertheless, because many adult NCDs have their biological and behavioural origins in childhood and adolescence, a substantial, if not fully quantifiable, proportion of this projected loss is traceable to early-life disease and risk-factor development. Such consequences stem from illnesses that disrupt educational attainment and cognitive development, thereby limiting future workforce participation [14–16]. Since many adult NCDs originate in early life, poor health during childhood and adolescence ultimately erodes long-term human capital formation [17].

This economic burden accelerates inequities, especially in low- and middle-income countries (LMICs) with fragile health systems and high out-of-pocket costs. Treatment for childhood cancers often consumes an entire household’s income, resulting in catastrophic health expenditures and treatment abandonment [18,19]. These financial shocks trap families in a multi-generational poverty cycle. By curtailing spending on nutrition and education, households inadvertently transmit health disadvantages to subsequent generations [20,21]. Beyond the domestic sphere, the unchecked rise of child and adolescent NCDs imposes substantial fiscal pressures on governments through the dual burden of escalating public health expenditures and a decline in tax revenue [22].

## SYSTEMIC BARRIERS TO INTEGRATED CARE FOR CHILDHOOD AND ADOLESCENT NCDs

The systemic bifurcation of health services actively obstructs the development of integrated chronic care models [9]. This is most critically observed in what may be termed the ‘transition gap’. As children with complex conditions age out of paediatric services, they enter a void where adult primary care is often ill-equipped or unfunded to receive them. Without integrated longitudinal records, these adolescents are frequently lost to follow-up [23], transforming manageable chronic conditions into acute clinical crises. While the evidence base for this phenomenon is most developed for type 1 diabetes and congenital heart disease, the absence of integrated longitudinal care pathways is common across the broader spectrum of childhood NCDs. Young people with juvenile idiopathic arthritis, childhood-onset mental health disorders (including eating disorders, ADHD, and early-onset psychosis), chronic kidney disease, and childhood cancer survivors all face structurally similar discontinuities as they transition into adult services. Evidence has reported poorer outcomes associated with transition failures, including increased hospitalisations among adolescents with sickle cell disease [24], reduced self-management and specialist engagement in asthma [25], increased risks regarding treatment late effects in childhood cancer survivors [26], and service disengagement among young people transitioning from child to adult mental healthcare [27]. This care vacuum is exacerbated by a critical deficit in specialised human resources. Across LMICs, the shortage of paediatric endocrinologists, cardiologists, and mental health specialists [28] forces a reliance on task shifting (delegating routine follow-up) and telemedicine. While these are necessary workarounds, quality assurance and equitable access remain inconsistent [29].

The lack of specialised care is a direct byproduct of a financing paradox. Global health funding remains dominated by ‘vertical’ programmes targeting HIV, tuberculosis, and malaria. Disease-based approaches continue to dominate the global health funding architecture, and despite the 2015 shift from the Millennium Development Goals to the Sustainable Development Goals (SDGs), funding priorities have been slow to follow [30]. Development assistance for NCDs, across all age groups, remained below 2.1% of total development assistance for health in 2022 [31]. Paediatric NCD financing is even less well tracked. It would be an overstatement to assert that vertical programmes mechanistically crowd out childhood NCD investment in all contexts; the empirical evidence on crowding-out effects in global health is mixed [32], but the pattern of concentrated political attention, institutional capacity, and funding in a narrow set of infectious diseases creates a structural opportunity cost that systematically deprioritises children's chronic conditions. This is compounded by the misconception that childhood and adolescent NCDs are rare [9]. Domestically, resources are disproportionately allocated to high-cost tertiary care, leaving community-based prevention and health promotion chronically under-resourced. These misallocations directly impact patients. Paediatric formulations are often unavailable, forcing clinicians to prescribe adult formulations off-label [33,34]. For impoverished families, high out-of-pocket costs render adherence an impossibility rather than a choice.

Sociocultural barriers further entrench these inequities. Stigma surrounding mental illness discourages adolescents from seeking care [35]. The ‘personal responsibility’ narrative surrounding obesity intensifies weight stigma, increases stress, discourages healthcare engagement, and exacerbates weight gain [36]. These dynamics are further compounded by surveillance gaps. While the GBD study and World Health Organization (WHO) Global Health Estimates [37] have generated important age-disaggregated data at the global level, children and adolescents remain systematically underrepresented in national surveillance systems. Conventional administrative age-grouping conventions, aggregating adolescents with children (0–14 years) or adults (15–34 years) [38], obscure condition-specific burden patterns and hinder evidence-based planning [39]. Disease registries, where they exist, are often limited to cancer or congenital anomalies, while information on metabolic or mental health conditions at the national level remains fragmented. These failures reflect a significant gap in global accountability mechanisms. Childhood and adolescent NCDs remain insufficiently represented in an adult-focused chronic illness discourse. The United Nations (UN) Political Declaration on Prevention and Control of NCDs [40] offers limited mentions of childhood interventions, and monitoring indicators continue to prioritise mid-life mortality.

## THE NEED FOR POLICY TRANSFORMATION THROUGH LIFE-COURSE AND SYSTEMS-THINKING

The life-course framework posits that health trajectories are determined by a continuum of biological, environmental, and social exposures beginning from the prenatal period [41]. Early-life adversities, ranging from maternal malnutrition and perinatal complications to environmental toxins and chronic psychosocial stress, are associated with increased NCD risks in later life [42,43]. The developmental origins of health and disease hypothesis provides a compelling theoretical basis for these associations, supported by a substantial body of both animal and human observational evidence. It is important, however, to acknowledge that the mechanistic evidence for epigenetic embedding in humans remains an active area of inquiry. Causal pathways are complex and context-dependent, and the field continues to advance [44,45]. Appropriate epistemic humility is warranted in translating these findings directly into policy claims.

Climate-related stressors act as a force multiplier in this process. They affect pregnant women in marginalised communities through multiple, partially overlapping mechanisms: heat-related physiological stress and placental dysfunction; food insecurity secondary to agricultural disruption; the foetal programming effects of particulate matter and ozone exposure; and psychological stress arising from climate-related displacement and insecurity [46]. These exposures increase the likelihood of preterm birth and low birth weight [47], both well-established antecedents in the developmental pathways leading to cardio-metabolic, respiratory, and neurodevelopmental NCDs. Additionally, climate-associated vector-borne and waterborne infections can trigger inflammatory and autoimmune responses that serve as NCD precursors, for example, through post-streptococcal pathways in rheumatic heart disease. The climate–gender–NCD nexus is therefore not merely conceptual. It operates through specific, biologically plausible mechanisms that warrant integration into both climate adaptation and NCD prevention strategies.

When this life-course perspective is combined with systems thinking, childhood and adolescent NCDs are understood as emerging from the nonlinear interactions of biological, behavioural, social, and economic subsystems [48]. Isolated clinical interventions are insufficient; they function only as reactive measures within a strained system. Sustained progress therefore requires a shift toward system-level policies that integrate education, urban planning, agriculture, and finance [9]. For example, Finland's universal school meal programme has demonstrated sustained improvements in children's diet quality through education-budget integration; Mexico's tiered taxation on sugar-sweetened beverages produced documented reductions in consumption, particularly among low-income households; Chile's front-of-pack warning label legislation has influenced both consumer purchasing behaviour and industry reformulation; and Thailand's Health Promotion Foundation (ThaiHealth) demonstrates how ring-fenced sin-tax revenues can fund sustained, multisectoral NCD prevention [49]. These examples are illustrative, not universally replicable: context-specific adaptation is essential. However, together they provide proof of concept that synergistic environments can alter the trajectory of childhood NCDs, and that investment in early-life prevention is an economic gain rather than a cost.

Despite these theoretical foundations, structural gaps persist. NCD prevention strategies remain primarily clinically oriented and neglect critical in utero and early-life determinants. This failure is most evident in pervasive ‘policy siloing’. Limited intersectoral cohesion and weak coordination result in fragmented policies across education, environment, and finance ministries, hindering the system-level coherence required to address social determinants of health. This fragmentation is further compounded by a chronic financing gap; prevention typically receives less than 3% of global health aid [50,51], although it should be noted that this figure is a global aggregate that masks inter-country variation. Some high-income countries with established public health infrastructure allocate a higher share, while in many LMICs prevention spending is even lower. Nevertheless, it serves as a globally significant benchmark for structural underinvestment in prevention relative to burden.

## THE ETHICAL IMPERATIVE OF ADDRESSING CHILDHOOD AND ADOLESCENT NCDs

The pervasive global neglect of child and adolescent NCDs represents a moral oversight in health governance. This neglect, rooted in what may be characterised as a moral hierarchy of disease, reflects a systemic bias that prioritises adult-centric illnesses or acute pathologies with immediate, visible mortality outcomes [37].

This neglect is empirically demonstrable across three domains: policy architecture, financing, and surveillance. The WHO NCD Global Monitoring Framework defines premature NCD mortality as the probability of dying between 30 and 70 years of life, thereby excluding all NCD deaths in children and adolescents from its primary target; national policies targeting youth risk factors remain limited in availability and scope, with substantial regional gaps [1]. Financing is similarly skewed: less than 2.3% of global development assistance for health is directed towards NCDs overall [51], and development assistance for child and adolescent mental health, the largest single NCD DALY contributor among those aged under 24 years, represented just 0.1% of total development assistance for health in developing countries over 2007–2015 [52]. Surveillance remains equally deficient: no systematic assessment of the adolescent NCD burden, its determinants, or the effectiveness of targeted interventions has been conducted [1], and existing tracking tools record only whether policies exist, without capturing data on implementation or impact.

This neglect is further compounded by commercial actors that actively construct environments normalising the consumption of ultra-processed foods, tobacco, alcohol, and digital-based products from an early age. Children are particularly vulnerable to these influences due to biological susceptibility, limited capacity for informed choice, and structural dependence on households, schools, and markets [12,39]. When examined through a child-rights and global health justice lens, profit-driven practices that externalise harm across the life course and reinforce intergenerational inequities raise profound ethical concerns. These concerns are grounded in empirical observation of these patterns, even as their normative implications necessarily rest on ethical reasoning rather than empirical data alone.

Addressing childhood NCDs and risk factors associated with future NCD development therefore requires recognising children as moral subjects with inherent rights. We argue that advancing health justice must go beyond distributive fairness to encompass two additional dimensions. Recognition justice [54] demands that children, especially those at the intersections of poverty and marginalisation, be acknowledged as legitimate stakeholders whose unique biological and social vulnerabilities actively shape global health priorities. In operational terms, this argues for the mandatory incorporation of child rights impact assessments into health policy planning; the inclusion of paediatric NCD indicators in UN Political Declaration monitoring frameworks; and disaggregated childhood NCD reporting within national health information systems. Procedural justice [55,56] demands the dismantling of consultative ‘tokenism’ in favour of inclusive governance structures that grant children meaningful agency in the policies that shape their futures. In practice, this may mean structured youth advisory panels within national NCD commissions; child-inclusive consultation standards in universal health coverage (UHC) benefit package design; and independent facilitation and feedback mechanisms that protect against unrepresentative or performative participation. We acknowledge the practical challenges posed by developmental variability in cognitive and deliberative capacities, the risk that participation mechanisms may disproportionately amplify the voices of urban or better-resourced youth, and the persistent institutional power asymmetries. These challenges argue for structural safeguards, rather than abandoning the principle of meaningful participation.

Embedding moral reasoning directly into health system design requires more than aspirational declarations. It requires accountability through global mechanisms to track progress on childhood NCD prevention and care, and through the recognition that governments, international agencies, and private actors bear shared, interdependent responsibility for shaping children’s health outcomes. Ultimately, the global health community is called upon to adopt an intergenerational ethic of care, one that views investment in children’s health as an ethical obligation to future societies.

## BREAKING INTERGENERATIONAL NCD INEQUITIES THROUGH STRATEGIC REORIENTATION

To move beyond the current clinical impasse, child and adolescent NCD prevention should be repositioned as a cornerstone of national development and fiscal stability. This requires a paradigm shift: transitioning from treating chronic illness as a medical cost to recognising it as a strategic investment in human capital [57]. Central to this integration is the expansion of UHC to include comprehensive child and adolescent NCD services within essential benefit packages. Fiscal policy should mirror this shift by institutionalising health-impact assessments, specifically cost-benefit analyses of early-life interventions. With evidence suggesting that every USD 1 invested in NCD prevention yields up to USD 7 in return [58], the economic ‘cost of inaction’ is no longer justifiable. Implementing progressive taxation on sugar-sweetened beverages and tobacco demonstrates both behavioural and fiscal efficacy. Ring-fencing these revenues specifically for child health programmes provides a model of sustainable, dedicated funding, as Thailand's ThaiHealth foundation shows [49].

Education policy integration is equally vital. Schools serve as unparalleled platforms for early detection, health literacy, and behavioural change [59]. Regular screenings, physical activity promotion, and nutrition monitoring must be embedded within education budgets rather than being treated as transient, externally funded health interventions [60].

**Figure 1:**
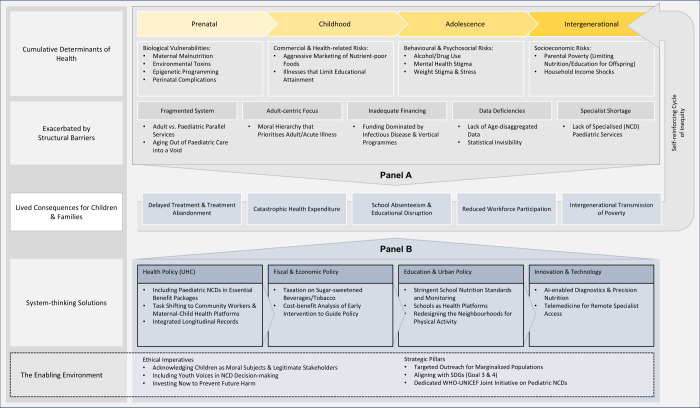
Integrated framework for breaking the cycle of inequity in child and adolescent NCDs. **Panel A.** Cycle of inequity. **Panel B.** Proposed systems transformation. Panel A delineates how cumulative biological, commercial, and social determinants of health interact across the life course from prenatal vulnerability to intergenerational transfer and are compounded by structural barriers, including adult-centric priorities, fragmented financing, and data invisibility. These systemic failures drive downstream consequences, including catastrophic household health expenditure, educational disruption, reduced workforce participation, and the intergenerational transmission of poverty. Panel B outlines a coordinated, multi-sectoral response grounded in systems thinking. It maps interventions across health, fiscal, educational, and technological sectors designed to dismantle structural barriers, interrupt the accumulation of harm across the life course, and prevent downstream social and economic consequences. The framework is anchored by an enabling foundation of ethical imperatives: recognition, procedural, and intergenerational justice, and strategic governance pillars that support equitable, sustainable action on child and adolescent NCDs.

A holistic vision for childhood and adolescent NCD prevention rests on three interlinked strategic pillars: equity, innovation, and sustainability (**Figure 1**). Equity is both a moral and policy imperative requiring interventions that effectively reach marginalised populations (including rural communities, refugees, and socioeconomically disadvantaged groups) who bear disproportionate NCD risks. This necessitates targeted outreach *via* community health workers and the horizontal integration of NCD screening into existing delivery systems, such as immunisation and maternal-child health programmes.

Innovation, both technological and financial, will shape the next generation of chronic disease prevention and care. Diagnostics enabled by artificial intelligence, wearable sensors for glucose monitoring or lung function, and precision nutrition approaches hold genuine transformative potential for early detection and self-management. However, several important equity risks should be acknowledged and actively mitigated. Tools supported by artificial intelligence are disproportionately developed using adults and high-income country data sets, generating risks of algorithmic bias and reduced diagnostic performance in paediatric and LMIC contexts. Wearable technologies and digital health platforms may preferentially benefit better-resourced populations, potentially widening the very inequities we seek to address. The deployment of these technologies should therefore be preceded by robust equity impact assessments, and be accompanied by strong ethical, regulatory, and data protection frameworks. Public sector investment in digital infrastructure in underserved settings is a prerequisite, not an afterthought. Blended financing models, including public–private and philanthropic partnerships, should be leveraged to scale innovations in resource-constrained settings, but only where equity safeguards are substantially built into governance structures.

Long-term sustainability demands the systemic embedding of childhood and adolescent NCD prevention within broader development and governance agendas. While the SDGs have provided an important normative framework, their limited progress highlights the inadequacy of aspirational targets devoid of accountability, financing, and structural reform. National development strategies should therefore move beyond symbolic alignment with SDG 3 (health) and SDG 4 (education) to explicitly incorporate measurable childhood and adolescent NCD indicators, with education recognised as a foundational social determinant of lifelong health.

The establishment of a WHO–UNICEF Joint Initiative on Paediatric NCDs [61] could serve as a catalytic coordination mechanism for funding mobilisation, data harmonisation, and policy coordination. We acknowledge that the global health governance landscape is already crowded, and that creating entirely new structures risks fragmentation and initiative fatigue. A more feasible initial approach may therefore be to establish this initiative as a standing technical working group within existing WHO–UNICEF structures, with a focused mandate to produce harmonised childhood NCD indicators, coordinate country-level capacity building, and produce high-level reporting into the UN General Assembly's NCD review cycles. Such an approach would parallel successful coordination models, including the Joint United Nations Programme on HIV/AIDS and the H6 Partnership for maternal, newborn, child and adolescent health, which have sustained political commitment through integrated monitoring and high-level reporting, without requiring entirely new institutional architectures. Protecting children and adolescents from preventable NCDs is ultimately a matter of intergenerational justice; equity for children must therefore become a central measure of societal progress.
